# Desquamative Gingivitis Revisited: A Narrative Review on Pathophysiology, Diagnostic Challenges, and Treatment

**DOI:** 10.3390/medicina61081483

**Published:** 2025-08-19

**Authors:** Doina Iulia Rotaru, Ioana Chifor Porumb, Lorentz Jäntschi, Radu Marcel Chisnoiu

**Affiliations:** 1 Department of Odontology, Endodontics and Oral Pathology, “Iuliu Hațieganu” University of Medicine and Pharmacy, 400012 Cluj-Napoca, Romania; doina.rotaru@umfcluj.ro (D.I.R.); marcel.chisnoiu@umfcluj.ro (R.M.C.); 2 Department of Preventive Dentistry, “Iuliu Hațieganu” University of Medicine and Pharmacy, 400012 Cluj-Napoca, Romania; ioana.chifor@umfcluj.ro; 3 Department of Physics and Chemistry, Technical University of Cluj-Napoca, 400641 Cluj-Napoca, Romania

**Keywords:** desquamative gingivitis, plaque control, oral health, corticosteroids, biopsy

## Abstract

*Background and objectives*: Desquamative gingivitis (DG) is a clinical term used to describe gingival conditions marked by erythema (unrelated to dental plaque), epithelial desquamation, and various intraoral lesions, with occasional extraoral involvement. It is typically linked to a range of underlying diseases. *Materials and methods*: A narrative literature review was conducted using PubMed, Scopus, Google Scholar, and the Cochrane Library, searching with keywords like “oral dysplasia”, “oral mucosa lesions”, or “desquamative gingivitis”. In addition to the literature review, a case report of a patient with DG is included to illustrate the diagnostic challenges and treatment considerations in a clinical setting, and to design and test simplified diagnosis and treatment-planning algorithms. *Results*: Diagnosis can be supported by a standard punch biopsy to obtain tissue samples for histopathological evaluation. The complex clinical case presented illustrates the clinical features of DG and highlights the challenges associated with its diagnosis and management. The mainstay of treatment, as resulted from 96 studies included in our review, involves topical and systemic corticosteroids, with topical calcineurin inhibitors serving as adjunctive therapy. *Conclusions*: A universally accepted treatment protocol is still lacking for DG, so this report outlines an effective, experience-based therapeutic approach. Additionally, it offers a simplified framework for diagnosis, treatment planning, and therapeutic management, contributing to the growing knowledge base needed for a decision-support algorithm development.

## 1. Introduction

Desquamative gingivitis (DG) presents a significant diagnostic and therapeutic challenge due to its diverse etiologies and variable clinical manifestations. The term “desquamative gingivitis” has been used since the early 20th century [1] to describe gingival conditions characterized by erythema, epithelial desquamation, and various intraoral lesions.

DG is a medical condition included among the clinical manifestation of immunologically mediated disorders which can affect the skin and mucosa [2,3]. Accurate diagnosis is essential to differentiate DG from conditions such as mucous membrane pemphigoid (MMP), intraoral pemphigus vulgaris (IPV), and oral lichen planus (OLP). DG is a relatively rare condition, which contributes to a lack of familiarity and expertise among clinicians.

Most of the DG cases were identified due to mucocutaneous conditions, later diagnosticated as oral lichen planus (OLP; 565 patients in [4], 271 in [5], 112 in [6], 41 in [7], etc.), mucous membrane pemphigoid (MMP; 121 patients in [8], 50 in [9], 25 in [10], etc.) and intraoral pemphigus vulgaris (IPV; 149 patients in [11], 28 in [12], 7 in [13], etc.). A retrospective analysis [14] of the relative incidence of OLP, MMP, and IPV has shown a OLP:MMP:IPV = 15:2:1 distribution.

Currently, there are neither validated diagnostic nor treatment algorithms for DG, thus further complicating clinical decision-making.

This narrative review aims to perform a survey on symptomatology and causes and a survey on epidemiological features of DG in order to provide an updated overview of the pathophysiology, diagnostic challenges, and treatment strategies for DG, and to report a series of observations in a case of DG, based on current scientific data and personal oral pathology clinical experience, with a focus on improving diagnostic accuracy and treatment outcomes. The guiding question of this article is whether the DG management can be improved and upgraded towards artificial intelligence-assisted diagnosis and management.

### 1.1. Background

#### 1.1.1. Clinical Features and Etiology

In many cases of DG, gingival lesions represent the onset of the vesiculobullous disorder and appear very early [15] during the clinical course. Most cases of OLP, MMP, and IPV start initially as gingival lesions [16].

OLP may occur alone or in combination with other skin forms of lichen planus. Its symptoms include patches of fine white lines and dots most commonly on the inside of the cheeks, gums, and/or tongue. The clinician should start identifying and removing any potential agent that might have caused a lichenoid reaction. Increased susceptibility is associated with [17] antibiotics, antimony, arsenic, chloroquine, diuretics, gold, iodides, phenothiazines, quinacrine, and quinidine. Careful consideration and targeted questions about any medication started in the last months, as well as any contact allergens identified by patch testing, are recommended.

MMP occurs both on the oral and on the ocular (scarring of the affected mucosa of the eye may lead to blindness) mucosa, but other sites that might be affected include the nasopharynx, larynx, genitalia, rectum, and esophagus; some environmental and genetic factors increase the susceptibility of its onset [18]. It is considered an autoimmune disease characterized by the production of autoantibodies against basement membrane zone antigens such as BP180, BP230, and laminin 5 [19].

IPV involves blistering and erosion of the skin, and mucous membranes primarily manifesting as soft blisters filled with clear fluid. Typical occurrences are blisters in the mouth (making it hard for the person to eat), followed by skin blisters that may come and go being associated with the binding of antibodies to the skin cells [20].

In the manifestation of DG, lupus erythematosus (LE), erythema multiforme (EM), epidermolysis bullosa (EB), epidermolysis bullosa acquisita, chronic ulcerative stomatitis, lichen planus pemphigoides, plasmacytosis, plasma cell gingivitis, orofacial granulomatosis, foreign body granulomas, graft-versus-host disease, and linear IgA disease can also be incriminated [21,22]. Exacerbations of DG have been associated with times of distress, anxiety, and mechanical trauma [23]. Dental plaque and calculus may also cause an exacerbation of DG [24].

In DG, the gingival condition can be easily characterized by painful erythema [25,26].

Because for most diseases the symptoms will vary from person to person, a useful resource is Human Phenotype Ontology (HPO), which is updated regularly. It may soon become a valuable resource for training models for AI-assisted diagnosis because it contains information on symptoms that have been described in medical resources [27], as well as the classification of systemic conditions’ gingival manifestations (Table 1).

#### 1.1.2. Epidemiological Features

DG is frequently seen in women around the age of 50, although rare cases have been observed in children. It is usually an indication of an oral or systemic disease [40,41,42,43,44]. Estimated incidence is summarized in Table 2.

The prevalence of DG is inconsistent in the scientific literature: a study including 414 patients with IPV, MMP, and OLP was found to be about 12% (49/414, [47]), but [48] the disease evolution is slow, and a delay of 19 months to diagnosis has been reported [45].

In some studies, MMP is counted as 35–60% of all cases of DG [49,50]; 24–45% of all cases of OLP; and 3–15% of IPV [50], MMP, IPV, and OLP, accounting for about 80% of the DG cases (majority are supported in [51]). The prevalence of DG in chronic ulcerative stomatitis, linear IgA disease, graft-versus-host disease, lupus erythematosus, epidermolysis bullosa acquisita, erythema multiforme, dermatitis herpetiformis, foreign body gingivitis, and plasma cell gingivitis was not determined.

## 2. Materials and Methods

A narrative review approach was chosen to provide a broad and contextualized understanding of this complex topic, drawing upon the authors’ expertise and a carefully curated selection of relevant literature.

### 2.1. Search Strategy

A literature search was conducted to identify relevant articles published between 1960 and 2025. The following databases were searched: PubMed, Scopus, Google Scholar, and the Cochrane Library. The search strategy used a combination of MeSH terms and free-text keywords to maximize the retrieval of the relevant literature. Examples of keyword included (“oral screening devices” or “autofluorescence” or “chemiluminescence” or “biopsy”) and (“oral dysplasia” or “oral mucosa lesions” or “desquamative gingivitis”). No language restrictions were applied initially, but only articles available in English, French, Hungarian, and Romanian were included in the final analysis.

### 2.2. Study Selection

The following inclusion criteria were applied to select articles for this review: (a) studies involving human subjects diagnosed with DG or related conditions (e.g., MMP, IPV, and OLP); (b) studies reporting on the etiology, pathogenesis, clinical features, diagnosis, or treatment of DG; and (c) original research articles, reviews, case reports, clinical guidelines, randomized clinical trials (RCTs), clinical trials, cohort studies, cross-sectional studies, case–control studies, pilot studies, and prospective and observational studies. A broad inclusion of recently diagnosed, untreated DG lesions was considered, while all participants in the studies were patients with suspicious oral lesions and/or a history of previously treated DG.

Exclusion criteria included the following: (a) studies not focused on DG or related conditions; (b) in vitro studies or animal studies; (c) studies with significant methodological limitations or biases; (d) duplicate publications; (e) lack of effective statistical analysis; and (f) abstract and author debates or editorials.

The study selection process involved screening titles and abstracts for relevance, followed by a full-text review of potentially eligible articles. Quality assessment of included studies was performed based on the reproducibility of the methodology and the results’ presentation.

### 2.3. Data Extraction

Data were extracted from the selected articles using a predefined data extraction form. The following data elements were extracted: study design, sample size, patient characteristics, diagnostic methods, treatment interventions, outcomes, and key findings. Data synthesis was performed using a narrative approach, with the aim of identifying common themes, patterns, and controversies in the literature.

Given the narrative nature of this review, a formal quality assessment of included studies was not performed. However, the authors considered the methodological rigor of each study when interpreting the results. Greater weight was given to findings from well-designed RCTs with larger sample sizes and clearly defined outcomes. Consistency of findings across multiple studies was also considered an indicator of data reliability. While this approach is inherently subjective, the authors strived to minimize bias by employing a comprehensive search strategy, applying consistent inclusion/exclusion criteria, consulting with experts in oral medicine, and presenting a balanced view of the evidence.

Data were collected directly from reports when available or from previous review studies. The authors, all having more than 20 years of clinical and research experience in general dentistry and oral pathology, independently extracted data from each report, but they supervised each other’s results in a rotational cycle (1 → 2 → 3 → 4 → 1). No automation tools were used. The PRISMA-style flow is displayed in Table 3.

This narrative review aimed to provide a broad synthesis of available studies, rather than a detailed methodological appraisal.

### 2.4. Case Report

Since DG is a rare condition, in addition to the literature review, a case report of a patient with DG is included to illustrate the diagnostic challenges and treatment considerations in a clinical setting, and to design and test simplified diagnosis and treatment planning algorithms. The case was conducted according to the guidelines of the Declaration of Helsinki and approved by the Ethics Committee of “Iuliu Hațieganu” University of Medicine and Pharmacy Cluj-Napoca, Romania (approval no. AVZ35/13.12.2021).

## 3. Results and Discussion

### 3.1. Clinical Features

**Figure 1:**
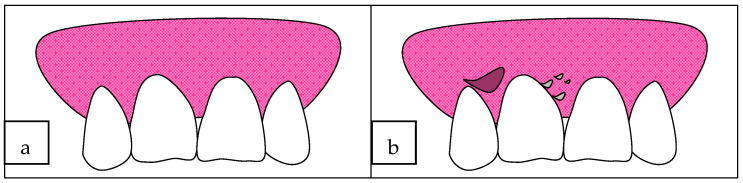

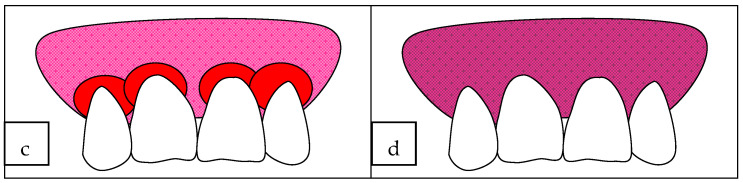
Clinical features in the context of DG onset: (**a**) normal gingiva (healthy coral pink color), (**b**) edematous gingiva with ulcerations, (**c**) bullous spreading all over the gingiva leaving ulcers, and (**d**) color changing to dusky red.

The clinical presentation of DG is polymorphous, and the presence of bacterial plaque can complicate the diagnosis. Most vesiculobullous diseases share similar clinical and microscopic features, desquamation, erosion of gingival epithelium and blister formation, other intraoral and sometimes extraoral lesions, and associated pain, especially after consumption of spicy foods. Nikolsky’s sign [52] often shows a positive reaction in patients with DG (the application of a shearing force on normal-appearing gingiva produces epithelial desquamation). Frequently, DG spares the marginal gingiva but can involve the whole thickness of the attached gingiva, and its clinical appearance is not greatly altered by traditional oral hygiene measures or conventional therapy alone [53]. Clinical features depend on the severity (Figure 1; desquamation and vesiculation of gingiva that becomes very fragile even under minimal pressure):

- The mild form, in which erythema is present, but this is painless;
- The moderate form, characterized by bright red and grey areas with patchy distribution, involving marginal and attached gingiva, smooth and shiny, slight pitting under pressure; also, massaging of the gingiva with the finger results in peeling of the epithelium and exposure of the bleeding connective tissue;
- The severe form, in which wide areas of the oral cavity are involved, surface epithelium appears shredded, and air-blowing causes a bubble in the gingival epithelium; it is painful and associated with a burning sensation.

Exacerbations of DG have been associated with periods of psychological stress, anxiety and mechanical trauma. Dental plaque and calculus may aggravate the DG.

### 3.2. Histological Analysis

Biopsy sampling should follow a standard procedure [54]. The sampling site should be in the interdental areas, avoiding the gingival margin by positioning the punch perpendicular to the lesion and performing simultaneous rotational movements under gentle pressure. The sample is placed onto a filter paper, with the mucosal surface turned upward to avoid curling or twisted artefacts, and it is introduced into a fixing solution of at least 10-fold the volume of the tissue sampled.

Direct immunofluorescence (DIF) testing and indirect immunofluorescence (IIF) testing are also indicated [55]. DIF is the gold standard used to diagnose many of the vesiculobullous diseases presenting as DG and is required for a definitive diagnosis [56].

The definitive diagnosis of DG is most accurately achieved when HE and two biopsies for DIF studies are submitted for testing, with DIF being a crucial diagnostic tool for differentiating DG-associated conditions. In MMP, DIF typically reveals linear deposition of IgG and/or C3 along the basement membrane zone, targeting antigens such as BP180 and laminin 5 [57]. IPV is characterized by intercellular deposition of IgG, primarily against desmoglein 3, within the epithelium [58,59]. While less specific, OLP may show fibrinogen or IgM deposition at the basement membrane zone but lacks the specific antibody patterns seen in MMP and IPV [60].

### 3.3. Diagnostic

The patient should be examined for intraoral and extraoral lesions for the identification of the affected area/areas. Gingival lesions, without other associated lesions—clinical suggestive for contact hypersensitivity reaction. They may be localized—most likely associated with dental restorative materials or generalized—and most likely caused by toothpaste or other substances containing flavoring agents or preservatives. In both cases biopsy is usually not necessary (if performed, it shows non-specific inflammation); DIF is negative. The clinician may consider allergy patch testing. Diagnosis is gingival contact hypersensitivity reaction, and the treatment is supposed to identify and remove the allergen.

### 3.4. Differential Diagnosis

Clinical, histological, and immunofluorescent findings in diseases causing desquamative gingivitis are non-specific [61], and often a differential diagnosis for OLP [62], IPV [58], and MMP [63] is necessary.

Even though a definitive diagnosis of the specific disease or disorder causing DG is required to provide proper treatment, it is almost impossible to differentiate between the diseases and disorders reported to result in DG based only on the clinical presentation.

Gingival ulceration is rare; it may cause severe gingival inflammation, discomfort, and bleeding, as the patients complain of “bleeding in the mouth” [64].

Differential diagnosis is strongly needed with cutaneous disorders and foreign body gingivitis (FBG). In cutaneous disorders, the skin lesions are missing, and the Nikolsky sign is negative. The histopathological examination reveals replacement of underlying connective tissue by a population of cells predominantly made up of plasma cells, characterized by foci containing particles of foreign material in the connective tissue that can have either a granulomatous or a lichenoid microscopic appearance. Usually, the lesions involve the marginal and attached gingiva; the interdental papillae are also commonly affected. The detection and elimination of the exposure to the etiologic antigenic agent will bring about the remission of the condition [65].

OLP, MMP, and PV are often associated with clinical signs of DG. Similar oral signs may occur in other pathologies, such as lichenoid reactions, systemic erythematous lupus, and erythema multiforme—which must be included in the differential diagnosis, as shown in Table 4, Table 5 and Table 6, which offer a summary of the relevant findings in 10 of studies included in our review.

An accurate diagnosis is important not only for treatment planning, but also for assessing the associated pathologies and the prognosis.

**Figure 2:**
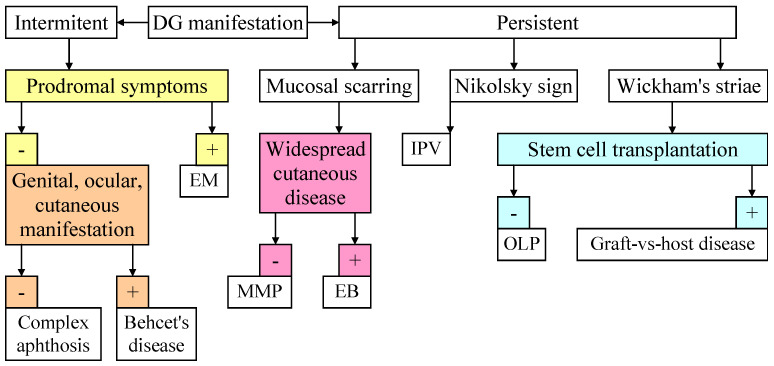
Clinical examination for DG: erythema multiforme (EM), mucous membrane pemphigoid (MMP), epidermolysis bullosa (EB), intraoral pemphigus vulgaris (IPV), and oral lichen planus (OLP).

Figure 2 provides an algorithm for managing a patient with DG.

### 3.5. Disease Management

The management of DG can be challenging for oral health practitioners. DG treatment continues to be a major problem, largely because the etiology of the disease is still elusive.

There is no standard treatment protocol available for the management of patients with DG. Key factors in determining treatment strategies include physician preference; patient age; the underlying disease causing DG; disease severity; presence or absence of extraoral lesions; and the patient’s medical history, including the systemic impact of the disease and potential complication from medications.

After successful diagnosis, the control phase is directed toward applying intense therapy to suppress the disease or disorder.

Essentially, the treatment of DG consists of the following [66]:

- Identification and elimination of the underlying cause whenever it is possible (avoidance of known/suspected allergens and irritants);
- Improvement of oral hygiene with consequent reduction of generalized plaque-induced inflammation and associated symptoms;
- Treatment of the underlying disease where available;
- Local or systemic immunosuppressive treatment (corticosteroids or other anti-inflammatory drugs);
- Other drugs: antimetabolites (cyclophosphamide, azathioprine, mycophenolate mofetil, and methotrexate), antibiotics (tetracyclines), dapsone, and immunoglobulins;
- Plasmapheresis;
- Surgery, gingival grafting, and/or laser therapy.

The exclusive gingival involvement in this multi-mucosal disorder entails careful history taking and diagnosis by a dental professional, thus signifying the role of dentists in such mucocutaneous disorders [67].

Treatment of DG requires elimination and/or control of local irritants. Improper restorations, bad-fitting dentures, dental plaque, traumatic oral hygiene procedures, and dysfunctional oral habits should be corrected [68].

It is demonstrated that gingival symptoms were improved by meticulous oral hygiene habits in some DG patients with OLP, and effective plaque control, but often they hinder from brushing their teeth due to pain and bleeding. Therefore, their oral hygiene is likely to be ineffective, making it difficult to treat this condition [69,70].

Topical corticosteroids (such as 0,25% desoximetasone, 0,20% fluocinolone, 0,05% fluocinonide, 0,5% triamcinolone acetonide, 0,05% betamethasone dipropionate, 0,05% clobetasol, and 0,05% halobetasol) and/or intralesional corticosteroids (triamcinolone acetonide) or other topical anti-inflammatory medication (topical tacrolimus, pimecrolimus, cyclosporine A, or tacrolimus rinse) can be effective.

Mild therapy (triamcinolone mixed in adherent paste or under an adhesive patch) may not have side effects, unlike prolonged therapy. A more aggressive therapy with high-potency or very high potency topical steroids and/or intralesional steroid injections is often recommended in severe lesions.

In patients with intense pain associated with DG, some authors [71] recommend using a viscous lidocaine gel in sealed trays for a few minutes (ten minutes), followed by the application of Perio Gel (5–15 min). Once the tissue health improves, a topical steroid gel is used in a tray (Lidex for 30 min, at least three times); this helps to control the severity of the DG, thus limiting the pain and destructive nature of this autoimmune syndrome. Changing medications at various times during the process also helps manage the elements of DG.

In cases with minimal response to topical therapy, systemic therapy should be considered. Clinicians should also be aware that DG may represent a lichenoid drug reaction, requiring identification and elimination of the causative agent.

Systemic corticosteroids used for short-term may be effective in rapid improvement of lesions, but they are more likely to produce adverse side effects.

Some authors treat DG lesions with topical corticosteroids when the systemically related disease is under control, and in cases of extraoral lesions, systemic steroids are the main choice [72].

Salivary cultures for candidiasis should be performed at baseline in patients with DG. If it is present, and if the diagnosis includes a condition requiring the use of topical or systemic steroids or other immune suppressants, prophylactic antifungal medication may be indicated at the onset of treatment. If this test is not available, the clinician should consider empirically prescribing an antifungal agent if immunosuppressants are to be used [73].

Since an appropriate level of control of the disease has been achieved, gradual tapering of medication should be initiated. The aim is to achieve complete remission or minimize symptoms and maintain this state of partial or complete remission on a long-term basis.

Petersen et al. suggested the use of fusidic acid cream in the treatment of DG, as it is known to exhibit antibacterial activity and has an important cytokine-suppressive effect [74].

Some DG lesions result from oxidative damage; therefore, antioxidants, which can delay or inhibit oxidation of oxidizable substrate in chain reaction, may be beneficial in treating DG. The antioxidant properties of honey were useful in the treatment of various vesiculobullous and DG lesions. Pure, unboiled, commercially available honey was topically applied. Excellent healing of DG lesions with no recurrence was recorded [75]. Other antioxidant agents have also been successfully tried for DG. Estrogen ointments, when applied topically, are effective in controlling DG. In refractory cases of DG, hormone replacement therapy (HRT) with low dose of estrogen is recommended, but only under the supervision of a physician [76].

Patients with DG should be monitored throughout their entire lives, considering the chronic nature of this disorder and the possibility of reactivation. It is recommended for the patients to be evaluated at 2 to 4 weeks after the beginning of the treatment until the condition is under control and they are free of symptoms. Further on, it is recommended to monitor patients every 3 to 6 months.

Every patient with DG is a specific entity and must receive individualized treatment, and dentists should be aware of signs of DG. Also, it may be necessary to have a management protocol that includes dentists/oral pathologists and dermatologists.

### 3.6. Treatment

Treatment of DG is differentiated according to diagnostic (Table 7).

**Figure 3:**
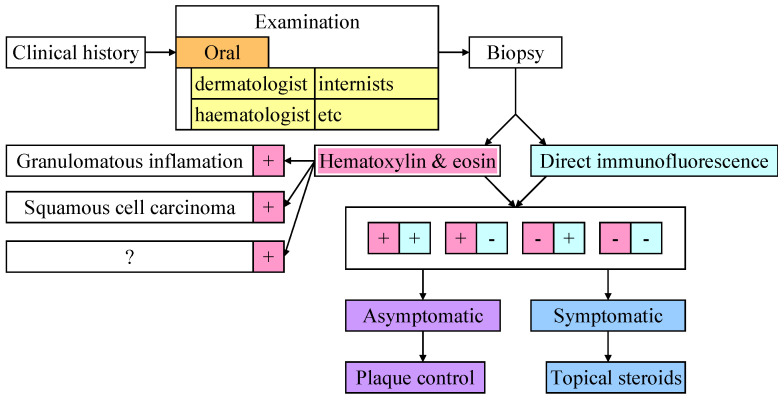
Simplified diagnosis and management of DG (“+” means visible changes; “-” means no pathological changes).

Regardless of the underlying condition, DG diagnosis and case management can be summarized according to the protocol described in Figure 3.

### 3.7. Case Report Results

**Figure 4:**
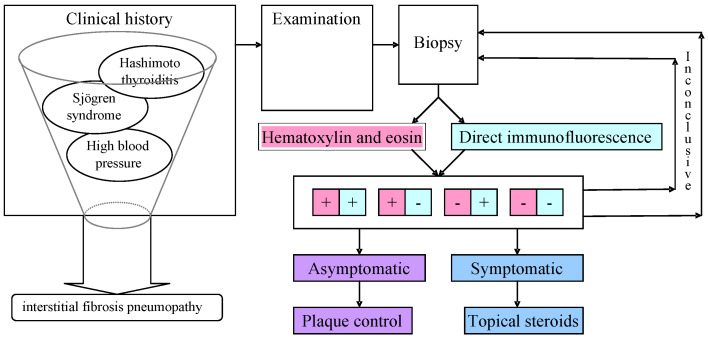
Patient P.I., 67-year-old woman, diagnosis and management algorithm.

Given the relatively low prevalence of DG in the general population [88], a detailed case report can offer valuable insights into diagnostic and therapeutic strategies. The following case presentation describes a 67-year-old female patient who presented to the Oral Pathology Department in “Iuliu Hatieganu” University of Medicine and Pharmacy with a four-year history of DG. This case was selected to illustrate how the application of simplified diagnostic algorithms could potentially enhance the efficiency and accuracy of clinical decision-making in similar cases (Figure 4), because, in this case, even the biopsies were inconclusive due to the variability of local conditions at the time of tissue preservation.

The chief complaint was intense erythema, desquamation and ulceration of the free gingival margin and extending to the attached gingiva, lips, and skin lesion on the arms, associated with pain.

**Figure 5:**
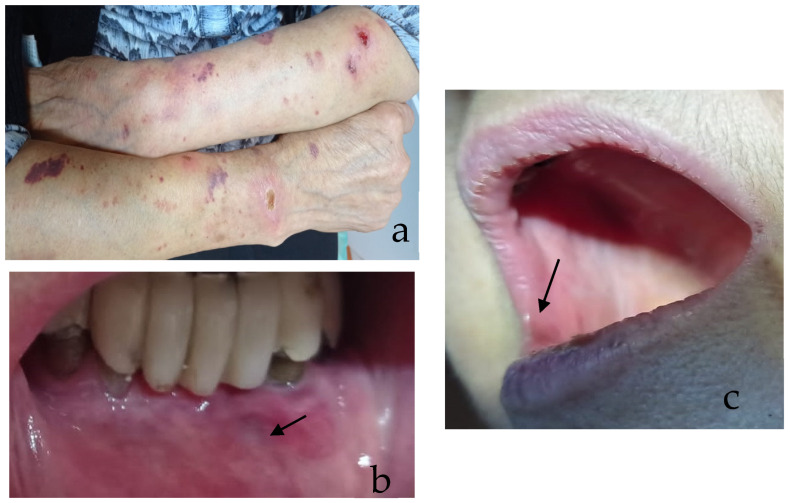
Clinical images of lesions in patient P.I.: (**a**) skin lesions located on the arms with plaque appearance, a glazed surface, and a lace-like patterning on the surface, slightly elevated; (**b**) erythematous erosive lip lesion (arrow); and (**c**) erythematous intra-oral lesions (arrow).

The patient’s clinical condition was suggestive for erosive oral lichen planus (Figure 5), even though the biopsies’ results were inconclusive.

Initially, the patient was administered topical corticosteroids, without any improvement of the symptomatology, continuing with systemic treatment both with corticosteroids and with Plaquenil, which unfortunately had important, almost intolerable side-effects, including occasional very high blood pressure episodes (>170 mmHg).

Although three biopsies were performed on different appointments, the diagnosis was still inconclusive. Moreover, the initial diagnosis of marginal chronical periodontitis, followed by periodontal regenerative surgery, worsened the patient’s condition. Because allergies were also considered, replacement of metal frameworks-fixed prosthesis with metal-free restorations was also performed, but the symptomatology was not significantly improved. Eventually, the only efficient intervention consisted in topical corticosteroids. This case could be an example of the importance of early topical therapy in DG; however, literature review-based diagnoses could become much more effective if they were computer-assisted by data input into complex decision algorithms and telemedicine remote expert opinion, because in most complex cases, such as in the above-presented one, the diagnosis remains inconclusive despite repeated biopsies.

This narrative review is limited by its subjective approach to literature selection and the potential for bias in the interpretation of studies.

## 4. Conclusions

DG presents diagnostic challenges for clinicians and pathologists, often leading to misdiagnosis and reduced quality of life. Currently, validated diagnostic algorithms for DG are lacking, and this review contributes to the systematization of foundational knowledge needed for their development.

## Figures and Tables

**Table 1:** Classification of systemic conditions’ gingival manifestations.

**Mucocutaneous Disorders ([28]):**	**Allergic Reactions:**
└OLP └MMP └IPV	└Dental materials [29] └Toothpastes [30] └Other additives (e.g., [31,32])
**Possibly drug induced ([33,34]):**	**Induced lesions:**
└EM [35] └LE [36]	└Drug-induced erosive lesions [37] └Sensitive skin and mucosa (e.g., EB [38])

Based on American Academy of Periodontology—2015 [39].

**Table 2:** Estimated incidences of the mucocutaneous disorders.

DG	Estimated Incidence
OLP	0.89% in the general population; 0.98% from clinical patients [41]
MMP	From 0.0001% to 0.0003% [42]
IPV	<0.0002% [43]
OLP:MMP:IPV	15:2:1 [12]; 15:15:1 [44]; 15:15:6 [45]; x:3:1 [46]

**Table 3:** PRISMA-style flow diagram.

Category	Number of Articles/Studies
Records identified	137
Duplicates removed	12
Records screened	125
Records excluded	16
Full-text articles assessed for eligibility	109
Full-text articles excluded	13
Studies included in the present review	96
Studies included in qualitative synthesis	26

**Table 4:** Immunologic findings of importance in the diagnosis of desquamative gingivitis.

Underlying Disease	Tissue: Pattern of Immune Deposits	Sera: Types of Antibodies
Cicatricial pemphigoid	Basement membrane zone (97%)	Basement membrane (23%)
Bullous pemphigoid	Basement membrane zone (100%)	Basement membrane (97%)
Pemphigus, all forms	Epithelial intercellular deposits (100%)	Intercellular antibodies of epithelium (over 95%)
Lichen planus	Globular deposits (cytoid bodies) in epidermis and dermis (97%) Fibrin deposits along basement membrane	None
Psoriasis	Stratum corneum deposits	None are specific for psoriasis
Other (hormonal, etc.)	Negative or few cytoid bodies	Negative

**Table 5:** Serology results’ overview for muco-dermal diseases.

	IgA	IgG	IgM	C3
Epidermal basal layer		X		X
Intraepithelial layer	X	X	X	X
Basement membrane		X	X	X
Epidermal–dermal border	X			
Papillary dermis	X		X	X
Blood vessel walls			X	X

**Table 6:** Overview of differential diagnosis in muco-dermal diseases.

Disease	Immunofluorescence Findings
Pemphigus vulgaris	IgG, IgA, and IgM in intraepithelial layer
Bullous pemphigoid	IgG and C3 in epidermal basal layer
Mucous membrane pemphigoid	IgG and C3 in epidermal basal layer
Paraneoplastic pemphigus	IgG and complement in intraepithelial layer and basement membrane
Linear IgA disease	Linear IgA along the epidermal–dermal border
Dermatitis herpetiformis	Granular IgA and complement in the tips of the papillary dermis
Chronic ulcerative stomatitis with stratified epithelial-specific antinuclear antibody	Speckled antinuclear antibody in lower third of epidermis
Lupus erythematosus	IgG and IgM in basement membrane
Erosive lichen planus	IgM in colloid bodies in papillary dermis
Erythema multiforme	IgM and C3 in blood vessel walls

**Table 7:** Treatment options for DG.

DG	Treatment
FBG	Periodontal: quadrant root planning under local anesthesia, followed by appropriate instruction about oral hygiene. Surgical: gingival grafts for the areas judged to be most receded and unstable, the lower canine and premolar buccal regions [77].
IPV	General principles of management and summary of treatment options for PV [78].
OLP	Topical and systemic medications used in the OLP treatment in [79].
MMP	Treatment algorithm (Behandlungsalgorithmus) in [80].
EM	Approaches to treatment, as in [81].
LE	Therapeutic options, as in [82].
EB	Ongoing interventional clinical trials, as in [83].
CUS	Doses of 200 mg hydroxychloroquine per day [84].
LIA	Therapeutic options, as in [85].
DH	Strict life-long gluten-free diet; wheat, rye, and barley are excluded from the diet, but the majority of patients with DH tolerate oats [86].
GvH	Topical therapies, including steroid mouthwashes, are recommended as first-line treatment. Extracorporeal photopheresis is recommended as a second-line systemic therapy for steroid-refractory GvH [87].

LE, lupus erythematosus; EB, epidermolysis bullosa; CUS, chronic ulcerative stomatitis; LIA, linear IgA disease; GvH, graft-versus-host disease; DH, dermatitis herpetiformis.
